# Supplementary material for: Mechanisms governing the pioneering and redistribution capabilities of the non-classical pioneer PU.1
Source: Nat Commun. 2020 Jan 21;11:402. doi: 10.1038/s41467-019-13960-2 (PMC6972792; doi:10.1038/s41467-019-13960-2)
Supplement: Supplementary file 7 — Source data [file 41467_2019_13960_MOESM7_ESM.zip › Source_Data/FigureS2/FigureS2B_MotifScanOutput/KmeansCluster2/homerResults/motif25.similar.html]

motif25

## Information for motif25

A
C
G
T
A
T
C
G
A
G
T
C
C
G
A
T
C
A
T
G
G
A
T
C
G
C
A
T
C
A
T
G
A
G
T
C
  
Reverse Opposite:  

T
C
A
G
G
T
A
C
C
G
T
A
C
T
A
G
G
T
A
C
G
C
T
A
T
C
A
G
T
A
G
C
T
G
C
A
  

|  |  |
| --- | --- |
| p-value: | 1e-49 |
| log p-value: | -1.148e+02 |
| Information Content per bp: | 1.587 |
| Number of Target Sequences with motif | 1478.0 |
| Percentage of Target Sequences with motif | 29.42% |
| Number of Background Sequences with motif | 8127.3 |
| Percentage of Background Sequences with motif | 19.99% |
| Average Position of motif in Targets | 101.0 +/- 54.9bp |
| Average Position of motif in Background | 100.9 +/- 73.0bp |
| Strand Bias (log2 ratio + to - strand density) | -0.0 |
| Multiplicity (# of sites on avg that occur together) | 1.15 |
| Motif File: | file (matrix) reverse opposite |

### Similar de novo motifs found

|  |  |  |  |  |  |  |  |
| --- | --- | --- | --- | --- | --- | --- | --- |
| Rank | Match Score | Redundant Motif | P-value | log P-value | % of Targets | % of Background | Motif file |
| 1 | 0.754 | A T G C G T C A T C G A A T G C C G T A A C T G A G T C C T G A | 1e-15 | -36.491733 | 10.41% | 7.05% | motif file (matrix) |
